# Identification of Host Proteins Interacting with IBV S1 Based on Tracheal Organ Culture

**DOI:** 10.3390/v15051216

**Published:** 2023-05-22

**Authors:** Huandong Zhang, Houli Cai, Qingyang Li, Chengxiu Fang, Li Peng, Jianing Lan, Jiyong Zhou, Min Liao

**Affiliations:** Key Laboratory of Animal Virology of Ministry of Agriculture, Zhejiang University, Hangzhou 310058, China

**Keywords:** infectious bronchitis virus (IBV), tracheal ring organ culture (TOC), virus-host interaction, heat shock protein 70 (HSP70)

## Abstract

Infectious bronchitis virus (IBV) belongs to the gamma-coronavirus genus of Coronaviridae and causes serious infectious diseases in the poultry industry. However, only a few IBV strains can infect avian passage cell lines, seriously hindering the progress of basic research on IBV pathogenesis. Whereas IBV field strains can replicate in tracheal ring organ culture (TOC) without any previous adaptation in chicken embryos or primary cells. In this study, to investigate the potential use of TOC as an in vitro infection model for the study of IBV-host interaction, we first established a chicken embryo TOC culture system and carried out an investigation on the IBV replication kinetics in the system. We found that the selected strains of the IBV GI-1, GI-7, GI-13, GI-19, and GI-22 genotypes could successfully replicate in TOC and bring about damage to the infected trachea. Next, we identified host proteins of the chicken embryo trachea that interact with the IBV S1 protein by immunoprecipitation and protein mass spectrometry. A total of 127 candidate proteins were initially identified with major involvement in cell adhesion pathways and apoptosis- and autophagy-related pathways. The heat shock protein 70 (HSP70) was selected for further investigation in the interaction with IBV viral proteins. Our results showed that HSP70 interacted with IBV S1 in both TOC and CEK cells, whereas HSP70 overexpression inhibited viral replication. This study indicates that TOC is a good system for the elucidation of IBV-host interactions and HSP70 is a potential host antiviral factor.

## 1. Introduction

Coronaviruses (CoVs) are classified into four genera based on phylogeny: alpha-CoV, beta-CoV, gamma-CoV, and delta-CoV. Avian infectious bronchitis virus (IBV) is a major species coronavirus in the gamma-CoV group, causing infectious bronchitis (IB) and impairing the respiratory, reproductive, digestive, and even nervous systems of poultry of all ages and different species [1]. Due to viral variations, vaccinations are not always effective. At present, IBV has spread worldwide, and IB is one of the most difficult-to-control infectious diseases in the poultry industry [2].

As one of the members of the CoVs, IBV possesses a single-stranded, positive-sense RNA genome, which comprises two overlapping open reading frames (ORFs) encoding polyproteins 1a and 1ab, the main structural proteins spike (S), envelope (E), membrane (M), and nucleocapsid (N). The viral S protein is the major surface protein of IBV, which is cleaved into S1 and S2 subunits by the host serine protease furin [3]. The S1 protein is associated with viral antigenicity and tissue tropism of the virus and plays vital roles in the induction of neutralizing antibodies and attachment to host cell receptors [3]. The interaction between viral and host proteins is an important means for viruses to successfully infect and replicate within host cells. However, few IBV strains can infect avian passage cell lines, which seriously hinders the progress of basic research on IBV pathogenesis.

Tracheal ring organ culture (TOC) is a commonly used and reliable method for isolating and culturing IBV, as an alternative to embryonated chicken eggs. IBV field isolates that do not replicate in primary cell culture can replicate and proliferate in TOC without previous adaptation in embryonated chicken eggs [4]. With the exception of a few reports using TOC as an in vitro model to study IBV pathogenicity [5,6], the application of TOC is limited in the isolation of field isolates. Therefore, it is of great significance to establish a TOC system to conduct host protein identification and IBV-host interactions.

The interaction between viral and host proteins is one of the most important routes for viruses to successfully infect host cells and carry out replication. Heat shock proteins (HSPs), including heat shock protein 70 (HSP70), are a large family of chaperone proteins found in most eukaryotes and bacteria. HSPs are responsible for the correct folding of proteins, protecting cells from stress, stimulating the production of immune and inflammatory factors, and regulating the host immune response. During the course of viral infection, it is utilized by the virus to complete the viral life cycle at all stages and to adapt to and escape the host’s antiviral response [7].

HSP70 is a receptor or coreceptor of various viruses, including dengue virus, Japanese encephalitis virus, and rotavirus [8,9,10]. To date, the functional cellular receptor for IBV infection is unknown. In addition, whether host HSP70 is associated with IBV infection awaits elucidation. In this study, HSP70 was found to be one of the predicted IBV S1-interacting candidates by LC-MS/MS analysis and was subjected to further confirmation.

## 2. Materials and Methods

### 2.1. Virus, Cells, Antibodies, Plasmids, and SPF Embryonated Chicken Eggs

IBV reference strains Beaudette (GI-1) and M41 (GI-1) as well as field isolates GX191219 (GI-7), QD191227 (GI-13), HF200702 (GI-19), and YC181031 (GI-22) used in this study were stored in our laboratory and were genotyped according to the typing method established by Valastro et al. [11]. Vero and 293T cells were maintained in DMEM supplemented with 10% FBS at 37 °C in a 5% CO_2_ atmosphere. Chicken embryonic kidney (CEK) cells were prepared as previously described [8]. In-house mAbs against IBV S1 (1H1), M (2B3) and N (4H4) proteins, and rabbit polyclonal antibodies against HSP70 were produced by our research group. Eukaryotic expression plasmids pCMV-Flag-N-HSP70 and pCMV-Myc-N-BD-S1were constructed by our group. Specific pathogen-free (SPF) embryonated chicken eggs were purchased from Ningbo Chunpai Agricultural Technology Company (Ningbo, China).

### 2.2. Preparation of Tracheal Ring Organ Culture

Tracheas were removed from 18 to 20 days old embryonated chicken eggs and placed into petri dishes containing DMEM. Fats and surrounding connective tissues were discarded as much as possible, and the lumen was rinsed 5–10 times using a syringe filled with DMEM. The cleaned tracheas were transversely sectioned into 0.5–1 mm tracheal rings with small sharp scissors. The rings were then washed twice with culture medium and placed into 24-well plates with 4 rings in each well containing 0.5 mL TOC medium (DMEM supplemented with 5% FBS and 0.1% antibiotic (containing 100 i.u./mL penicillin and 100 ig/mL streptomycin)) and cultured in a 37 ℃ cell incubator with 5% CO_2_. After culturing for 12–24 h, tracheal rings with active ciliary beating were used for infection.

### 2.3. Determination of TOC-ID_50_ of IBV Viruses

The TOC-ID_50_ of the IBV virus strain was measured using tracheal rings with vigorous beating cilia after a 24 h preparation. Serial dilutions of IBV-infected chicken embryo allantoic fluid ranging from 10^−1^ to 10^−10^ were inoculated into the tracheal rings in the wells of the 24-well plates, with 5 wells for each dilution, followed by incubation at 37 °C with 5% CO_2_ for 1 h. Next, after 3 washes with PBS, the tracheal rings were cultured with TOC medium in the incubator for 144 h. During this period, the culture medium was renewed every 48 h. The samples were scored as infection-positive when 70% ciliostasis was observed. The calculation of TOC-ID_50_ was conducted according to the Reed–Muench method as follows: distance ratio = (positive rate above 50% − 50%)/(positive rate above 50% − positive rate below 50%) and lgTOC-ID_50_ = logarithm of the highest antibody dilution above 50% positive rate + distance ratio × logarithm of the dilution factor.

### 2.4. Infection of TOC with IBV

Freshly prepared TOCs with vigorously beating cilia were infected with different IBV strains at a dose of 1000 TOC-ID_50,_ and uninfected TOCs were used as controls. Successful infection was confirmed by RT-PCR using one set of primers targeting the IBV N gene (IBV-N-F: 5′-GATGGTAATTTCCGTTGGGA-3′/IBV-N-R: 5′-CATTGTTCCTCTCCTCATCT-3′). Briefly, 3 tracheal rings in 500 μL PBS were processed using a tissue crusher for 1 min, followed by 3 rounds of repeated freezing and thawing, and the supernatant was subjected to total RNA extraction using TRIzol reagent. The RNA was then transcribed into cDNA according to the instructions provided by the Reverse Transcription Kit (Thermo Fisher Scientific, Waltham, MA, USA). PCR was performed in a 25 μL reaction containing 12.5 μL of 2× PCR mixture, 0.5 μL of each primer (10 μM), and 1.5 μL of cDNA (10 ng/μL), then subjected to the amplification programs at 95 °C for 5 min, 35 cycles of denaturation at 94 °C for 30 s, annealing at 50 °C for 30 s, an extension at 72 °C for 30 s, and a final extension of 72 °C for 10 min. Real-time qRCR (RT-qPCR) was performed to detect the relative abundance of IBV-N transcripts using the ChamQ Universal SYBR qPCR master mix (Vazyme Biotechnology, Nanjing, China) in a LightCycler 96 sequence detector system (Roche Diagnostics, Mannheim, Germany). A grading system of 0 (100% ciliostasis) to 4 (0% ciliostasis) was used to score the ciliary activity of the TOC. The activity of control tracheal rings at 12 hpi was recorded at the highest level of “4”, whereas the scores in the other groups ranged from 0 to 4 at the different infection time points, respectively. For further analysis, the dynamics of IBV infection in TOC were monitored via 6 samples for each time point with 3 rings for HE, IHC, and IFA assays, with the remaining 3 were used for Western blot analysis.

### 2.5. HE, IHC and IFA

Three tracheal rings in each tube were washed once with 4% paraformaldehyde (PFA), then kept in 1 mL 4% PFA and sent to Nanjing Freethinking Biotechnology Co., Ltd. (Nanjing, China) to perform hematoxylin-eosin (HE) staining and immunohistochemistry (IHC) assay. 

### 2.6. Immunoprecipitation and Co-Immunoprecipitation Assays

The membrane proteins from IBV YC181031 infected TOCs were extracted using the Membrane Protein Extraction Kit (Beyotime, Shanghai, China) following the manufacturer’s protocol. Membrane protein extracts were immunoprecipitated (IP) with mAb 1H1 against IBV S1 protein and a mAb against mouse IgG (A7028, Abcam, Cambridge, UK). The precipitated proteins were detected by silver staining and Western blot analyses using anti-S1 and anti-HSP70 antibodies. Next, 293T cells were cotransfected with pCMV-Flag-N-HSP70/Myc-N-BD-S1, and the Flag-tagged HSP70 was immunoprecipitated with Flag antibodies and captured by Sepharose A/G beads. The precipitated proteins were then detected by Western blot analysis using anti-Flag antibodies and mAb 1H1 to IBV S1.

### 2.7. SDS-PAGE, Western Blot Analysis and Silver Staining

TOC lysates and immunoprecipitated proteins were separated using SDS-PAGE and transferred onto a nitrocellulose membrane. After blocking with 5% skim milk, the membranes were incubated with the indicated primary antibodies at 4 °C overnight. After three washes with PBS, the blots were incubated with HRP-labeled anti-mouse/rabbit IgG at 4 °C for 1 h. Protein bands were then visualized using an enhanced chemiluminescence reagent and imaged using an AI680 Imager (GE Healthcare, Chicago, IL, USA).

The polyacrylamide gel was fixed with a 4:1:5 ratio of ethanol, glacial acetic acid, and water at room temperature for 2 h and then washed thrice with water. After incubation with a sensitizing solution containing sodium acetate, sodium thiosulfate, and absolute ethanol, the gel was transferred to the silver stain solution and incubated in the dark at room temperature for 30 min. Finally, a color developer containing anhydrous sodium carbonate, formaldehyde, and sodium thiosulfate was used and incubated in the dark with the gel. The reaction was terminated by adding 5% glacial acetic acid. The stained bands were imaged using an ImageScanner III instrument, and the bands of interest were excised from the gel for liquid chromatography-mass spectrometry (LC-MS) analysis.

### 2.8. Mass Spectrometry and Analysis

Three independent immunoprecipitation experiments to identify the host proteins interacting with IBV S1 were performed. After silver staining detection, specific bands in the IBV S1 mAb group as compared to the control IgG group were collected from three independent gels and sent for commercial mass spectrometry (LC-MS/MS) analysis carried out by Shanghai Applied Protein Technology Company. Protein searches were carried out using Mascot 2.2 software. Protein identification was performed using the following criteria: (a) trypsin-digested peptides with 2 max missed cleavages allowed, (b) proteomics tools: 3.1.6, (c) >1 unique peptides, (d) filter by score ≥20. Proteins found in the respective negative control samples were eliminated from the dataset to remove nonspecific interactions. Proteins represented by at least two unique peptides were considered for further analysis, and the proteins in the negative control were excluded.

Candidate proteins from LC–MS/MS analysis were subjected to GO annotation and KEGG analysis by using the DAVID website (https://david.ncifcrf.gov/home.jsp, accessed on 17 March 2021) under the three major categories of biological processes (BO), molecular functions (MF), and cell components (CC).

### 2.9. Infection of Vero Cells Overexpressing HSP70 with the IBV Beaudette Strain

The plasmid pCMV-Flag-N-HSP70 was transfected into Vero cells using the jetPRIME kit (Polyplus). The cells were infected with the IBV Beaudette strain (MOI = 1) 24 h after transfection and incubated at 37 °C for 12 h, 24 h, and 36 h, respectively. The cells were harvested after three washes with PBS and subjected to Western blot analysis.

## 3. Results

### 3.1. Infection of TOCs with IBV Strains

The ciliary activity of TOCs was inhibited during preparation but recovered gradually upon culturing at 37 °C. After 24 h, the ciliary activity of TOCs remained at a high level and could be used to determine virus TOC-ID_50_ values and IBV infection. As calculated by the Reed–Muench method, the TOC-ID_50_ values of the M41 reference strain and the GX191219, QD191227, HF200702, and YC181031 field isolates were 10^−5.0^/mL, 10^−5.8^/mL, 10^−6.56^/mL, 10^−6.77^/mL, and 10^−7.5^/mL, respectively.

**Figure 1:**
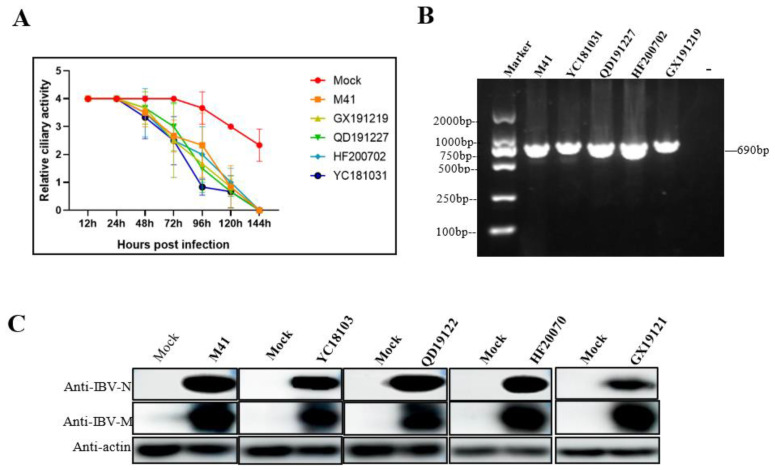
Infection of TOCs with IBV strains. (**A**) Ciliary activity of IBV-infected TOCs. TOCs were infected with 1000 TOC-ID_50_ of selected IBV strains or mock infected with medium. Ciliary activity was assessed under a light microscope at 12, 24, 48, 72, 96, 120, and 144 hpi and scored as follows: 100% activity = 4, 75% = 3, 50% = 2, 25% = 1, and 0% = 0. Data represent the mean of triplicate wells, with error bars showing the standard error. (**B**) Detection of IBV in infected TOCs by RT-PCR. The TOCs infected with selected IBV for 36 h were collected and subjected to RT-PCR amplification with primers targeted to the IBV N gene. (**C**) Detection of IBV in infected TOCs by Western blot analysis. The TOCs infected with selected IBV for 36 h were collected and subjected to protein sample preparation followed by Western blot analysis with mAbs to IBV N and M.

Five selected IBV strains including M41 and four field isolates (GX191219, QD191227, HF200702, and YC181031) at a dose of 1000 TOC-ID_50_ were used to infect tracheal ring samples, and ciliostasis of the TOCs was observed under a microscope over a 144-h period. As shown in Figure 1A, while ciliostasis was unobserved after 24 h post infection (hpi), ciliary activity dramatically decreased from 48 hpi to 96 hpi for the five selected strain infections compared to the mock. Complete ciliostasis was observed at 144 hpi (Figure 1A). A specific IBV N gene fragment of around 690 bp was amplified from all the IBV-infected TOCs (Figure 1B), which was further confirmed by sequencing, indicating that the five IBV strains were successfully inoculated into TOC culture. In addition, both the M and N viral proteins were detected by Western blot analysis (Figure 1C), indicating successful infection of TOCs with selected IBV strains.

**Figure 2:**
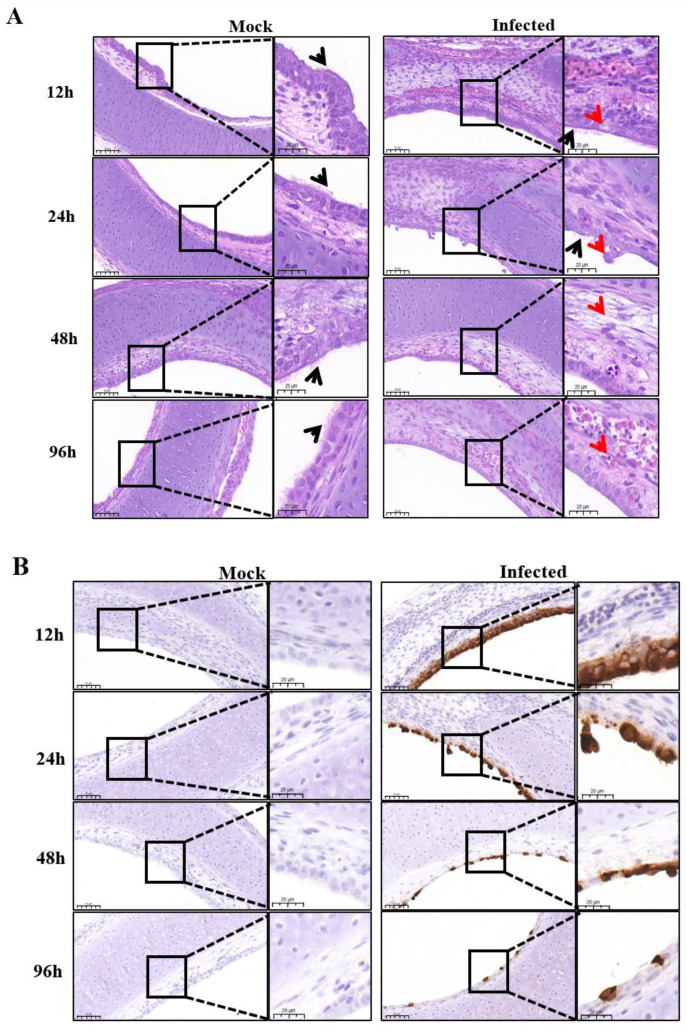

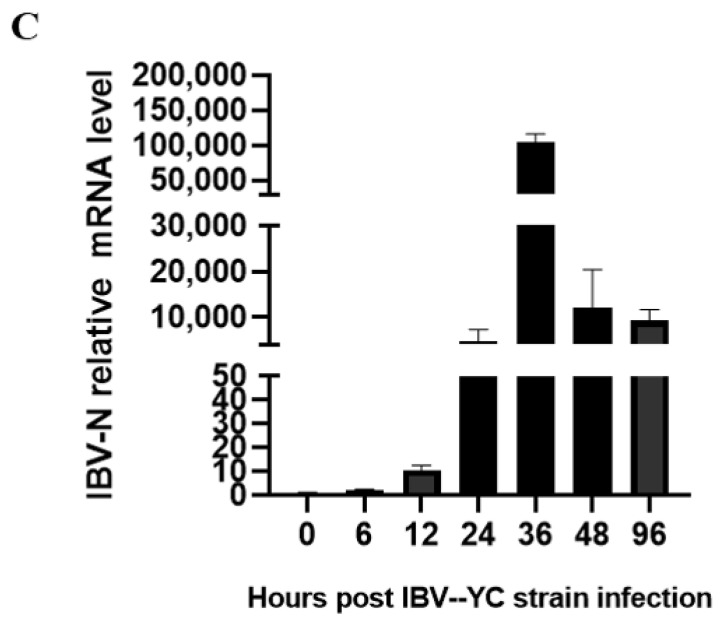
Investigation of histological lesions and detection of viral replication in IBV-infected TOCs. (**A**) Histopathological changes of IBV-infected TOCs at 12, 24, 48 and 96 hpi by hematoxylin-eosin staining. Cilia of TOCs was indicated by black arrows. Red arrows indicate degenerated epithelial cells at 12 hpi, exfoliation of epithelial cells at 24 hpi, edema of the lamina propria at 48 hpi and lymphocyte infiltration at 96 hpi, respectively. Scale bars in low and high magnification image are 50 μm and 20 μm, respevtivel. (**B**) immunohistochemistry examinations of IBV-infected TOCs at 12, 24, 48 and 96 hpi using mAb to IBV S1. Scale bars in low and high magnification image are 50 μm and 20 μm, respevtivel. (**C**) Detection of viral replication in IBV-infected TOCs at 0, 6, 12, 24, 36, 48 and 96 hpi by RT-qPCR.

To further investigate the histological lesions imposed on the trachea upon IBV infection, HE staining was performed on IBV YC181031 infected TOCs at different infection time points. Histopathological examination revealed that the epithelial cells of the tracheas in the infected group suffered degeneration, with increased intracellular vacuoles observed at 12 hpi. At 24 hpi, the morphology of the epithelial cells of the infected TOCs was not as clear as that of the control group, and necrosis and exfoliation of epithelial cells were observed in the infected group. Cilia in the infected tracheas were lessened at 24 hpi and completely disappeared by 96 hpi; edema of the lamina propria occurred by 48 hpi and lymphocyte infiltration was noticed at 96 hpi in infected group (Figure 2A). In contrast, cilia of uninfected TOCs could be clearly observed over the entire 96 h period (Figure 2A). The maximum number of epithelial cells of IBV-infected TOCs were observed at 12 hpi by IHC and gradually decreased as infection progressed, with only very few cells harboring viral signals observed at 96 hpi in the examination (Figure 2B). Viral replication peaked at 36 hpi, as indicated by RT-qPCR (Figure 2C).

### 3.2. Identification of S1-Host Interactions in the IBV-Infected TOCs

**Figure 3:**
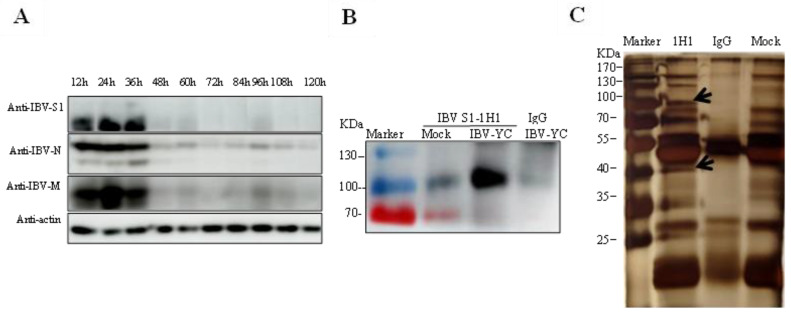
Identification of IBV S1 interacting host proteins from TOCs. (**A**) Determination of viral protein expression levels in TOCs infected with IBV YC181031. TOCs were infected with 1000 TOC-ID_50_ of YC181031 in 24-well plates. Infected TOCs were harvested at 12, 24, 36, 48, 60, 72, 84, 96, 108, and 120 hpi. The supernatant of TOC lysates was subjected to SDS-PAGE and Western blot analysis with mAbs to S1, N, and M proteins. The expression of cellular actin protein was used as a control. (**B**) Western blot analysis of immunoprecipitated proteins using mAb 1H1 against the IBV S1 protein. Mouse IgG was used as a control. The arrow indicates the S1 protein. (**C**) Silver staining of immunoprecipitated proteins. The supernatants of TOC lysates were immunoprecipitated against mAb 1H1 to the IBV S1 protein and mouse IgG, respectively. Uninfected TOCs were used as mock controls. Arrows indicate the protein bands at 100 kDa and 40 kDa, specifically precipitated by mAb 1H1.

Thus far, the TOC approach has rarely been used in the study of IBV-host interactions. To identify potential host proteins binding to IBV S1 in the TOC, we first checked viral protein expression during infection. As shown by Western blot analysis, the expression level of S1, N, and M proteins of IBV YC181031 in TOC peaked at 24–36 hpi (Figure 3A); therefore, this period was selected as the best time point for the preparation of immunoprecipitated S1 protein in the following experiments. 

The membrane proteins obtained from the lysate of TOC infected with YC181031 at 36 hpi were extracted and subjected to immunoprecipitation assays with mAb 1H1 against IBV S1 protein and anti-mouse IgG. IBV S1 subunits around 100 kDa from the immunoprecipitated fragment were detected by Western blot analysis with mAb 1H1 to IBV S1, but not detected in control IgG (Figure 3B). Two specific protein bands with molecular weights of around 100 kDa and 40 kDa (arrows) were visualized by silver staining in 1H1 immunoprecipitates from infected TOCs, but were absent in control IgG and mock immunoprecipitates (Figure 3C). The two specific bands in 1H1 immunoprecipitates as well as the corresponding fragments in control IgG and mock immunoprecipitates were excised, respectively, and sent for LC-MS/MS analysis. A total of 127 potential IBV S1-interacting host proteins were identified (Supplementary Table S1), which were absent from the data from control groups.

**Figure 4:**
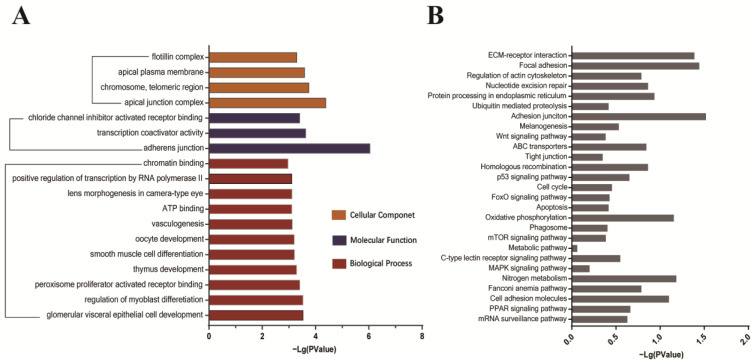
Gene ontology and KEGG analysis of the identified IBV S1-host interactome. (**A**) Gene ontology analysis of potential interacting host proteins. Significantly enriched terms were identified based on biological process (BP), molecular function (MF), cellular component (CC), and *p*-value < 0.05. (**B**) KEGG pathway enrichment analysis.

To establish the host cell proteins or pathways that have been enriched in the IBV S1-TOC protein interaction network, we performed gene annotation and analysis on the biological processes, molecular functions, and cell components of the candidate proteins. A large number of biological processes, such as glomerular visceral epithelial cell development, regulation of myoblast differentiation, and peroxisome-proliferator activated receptor binding, were enriched. In addition, adherens junction, transcriptional coactivator activity, and chloride channel inhibitor activity were enriched under molecular functional categories, whereas apical junction complexes, chromosome telomere regions, apical plasma membranes, and flotillin complexes were enriched under cell component classifications (Figure 4A, Supplementary Table S2).

In order to further predict the cellular metabolic and signal transduction pathways of candidate host proteins interacting with IBV S1 protein, we conducted a pathway enrichment analysis using the KEGG database. The pathways targeted by the candidate proteins analyzed by KEGG are presented in Figure 4B and Supplementary Table S3. Interestingly, the majority of the target proteins were involved in extracellular matrix (ECM) receptor interaction, local adhesion, adhesive binding, cell adhesion molecules, and C-type lectin receptor, which might be important for IBV invasion. In addition, there were some candidate proteins participating in ubiquitin-mediated proteolysis, the p53 signaling pathway, the phagosomes, the mTOR signaling pathway, and the MAPK signaling pathway, which are involved in immune response, autophagy, and apoptosis (Figure 4B, Supplementary Table S3).

### 3.3. Confirmation of the Interactions between IBV S1 and HSP70 in Both Transfected Cells and TOCs

**Figure 5:**
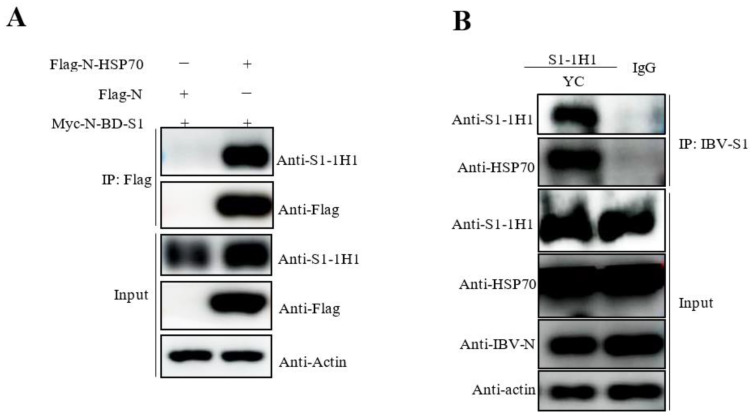
Confirmation of the interactions between IBV S1 and HSP70 by immunoprecipitation assay using mAb 1H1. (**A**) Exogenous HSP70 interacts with IBV S1 in transfected cells. The 293T cells were cotransfected with the plasmids pCMV-Flag-N-HSP70 and pCMV-Myc-N-S1 for 36 h. The input protein complexes and immunoprecipitated proteins were stained with antibodies against S1, Flag, or Actin. (**B**) Endogenous HSP70 interacts with IBV S1 in IBV YC181031 infected TOCs. The TOCs were infected with 1000 TOC-ID_50_ for 36 h. The input protein complexes and immunoprecipitated proteins were stained with antibodies against S1, N, HSP70, or Actin.

Immunoprecipitation assay was performed with mAb 1H1 against the IBV S1 protein in S1 and HSP70 cotransfected 293T cells and YC181031 infected TOCs. As shown in Figure 5A, HSP70 could be immunoprecipitated by mAb against the IBV S1 protein from HSP70 transfected 293T cells but not from control vector-transfected cells, indicating that exogenous HSP70 could interact with IBV S1 (Figure 5A). In the endogenous immunoprecipitation assay, host HSP70 was immunoprecipitated by a mAb to IBV S1 from infected TOCs, but not by mouse IgG (Figure 5B).

### 3.4. Effects of HSP70 on the Replication of IBV Beaudette Strain in Vero Cells

**Figure 6:**
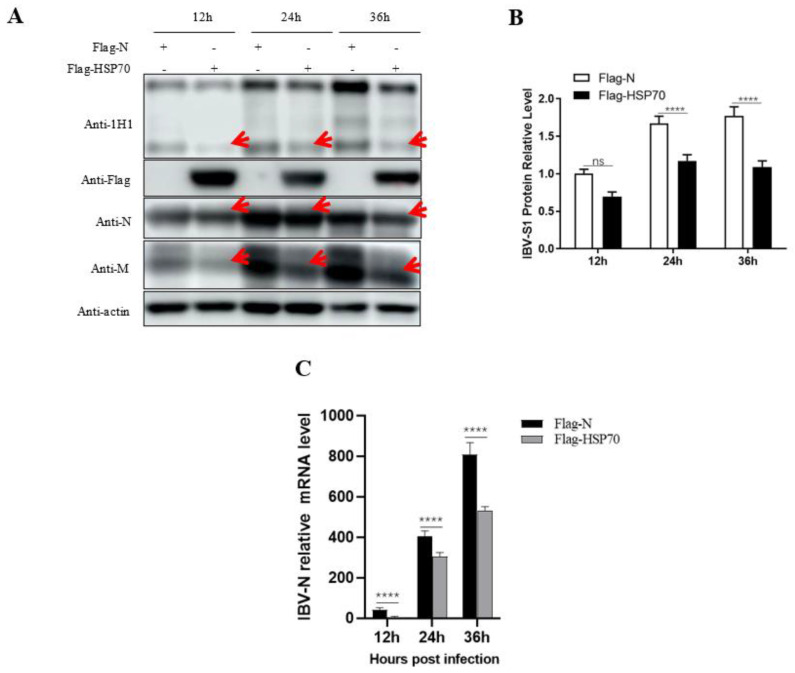
Effects of HSP70 on the replication of IBV Beaudette strain in Vero cells. (**A**) The pCMV-Flag-N-HSP70 plasmid was transfected into Vero cells following infection with the IBV Beaudette strain (MOI = 1) 24 h after transfection and incubated at 37 °C for 12 h, 24 h, and 36 h. The cells were harvested and subjected to Western blot analysis with the indicated mAbs to IBV S1, M and N and mAbs to Flag and actin. Arrows indicate reduced expression of IBV S1, N, and M proteins, respectively. (**B**) Intensity analysis of the S1 protein bands in panel A. Data were presented as means ± SD of three independent biological experiments. ns, not significant, *p* > 0.05; **** *p* < 0.01. (**C**) Detection of virus replication in HSP70 overexpression Vero cells infected with Beaudette at 12, 24, and 36 hpi by RT-qPCR. The data were analyzed using the 2^−∆∆CT^ method and are presented as means ± SD of three independent biological experiments. **** *p* < 0.01.

To preliminarily understand the effects of HSP70 on IBV replication, the IBV Beaudette strain was used to infect HSP70-overexpressing Vero cells. Infected cells were collected at 12, 24, and 36 hpi and subjected to Western blot analysis and RT-qPCR. As shown in Figure 6A,B, the expression levels of viral S1, N, and M proteins were significantly reduced in HSP70 transfected cells at all the indicated time points compared to the control vector-transfected control groups (Figure 6A,B). The mRNA relative levels of S1 in HSP70 transfected cells were also lower than those of the control vector-transfected control groups at all the time points (Figure 6C).

## 4. Discussion

In this study, one IBV reference strain M41 and four isolate strains (GX191219, QD191227, HF200702, and YC181031) were used to infect the tracheal rings of chicken embryos, respectively. The degree of ciliostasis was used to evaluate the activity of tracheal tissues infected with IBV [4]. All selected strains could cause ciliostasis without any previous adaptation. Cilial activity of the tracheas in all infected groups maintained for 2 days after infection, then decreased at 2–4 hpi accompanied by gradual loss of cilia, followed by complete ciliostasis at 144 hpi (Figure 1A). The ciliary activity of YC101031 infected tracheas seemed to decrease more at 96 hpi (Figure 1A) than those in the other infected groups, suggesting different susceptibilities among strains towards tracheal damage. We did not perform a pathogenicity comparison study among the selected strains. In a previous study, we found that the vaccine strain H120 causes complete ciliostasis 72 hpi earlier than that of some field isolates. Therefore, it is difficult to comment on whether the degree of damage to tracheal rings by the virus is related to its virulence. This requires more experimental data.

The selected virus replicated in epithelial cells very well. As shown by IHC analysis, almost every epithelial cell was infected by the virus at 12 hpi (Figure 2B), while ciliary beating was still very active at 12 hpi, as observed with microscopy (Figure 1A). In this case, the detection of IBV infection by IHC was more sensitive than ciliostasis observation. Microscopic observation revealed that the cilia of the tracheal ring remained active 24 h after infection (Figure 1A). However, at this time point, some epithelial cells dropped off the rings, leading to a decrease of virus staining cells in the infected trachea at 24 hpi as compared to that at 12 hpi, as detected by IHC (Figure 2B). As infection progressed, more ciliated epithelial cells of the tracheas became necrotic and shed from the tracheas at 48 hpi, and by 96 hpi, only a few infected epithelial cells remained, with cilia completely disappearing (Figure 2A,B).

Using IHC analysis, we clearly noticed epithelial cells stained by IBV mAb at 12 hpi, after which the number of stained cells decreased (Figure 2B). However, by RT-qPCR (Figure 2C) and Western blot (Figure 3A) analysis, the highest mRNA expression level and highest protein expression level were observed at 36 hpi and at 24–36 hpi, respectively (Figure 2C and Figure 3A). We think this discrepancy is due to the infected cells dropping off the tracheas gradually, but the epithelial cells that had dropped off were collected and subjected to the Western blot and RT-qPCR analysis. Therefore, the TOCs infected at 36 hpi were selected for the study of virus-host interactions by immunoprecipitation.

GO and KEGG analyses were performed on the identified candidate proteins. Enrichment analysis results showed that the host proteins interacting with the S1 protein are closely related to cell adhesion and connection, protein metabolism, and transport processes and participate in receptor interaction, apoptosis, and autophagy signaling pathways (Figure 4A,B and Supplementary Tables S2 and S3). Among the identified candidates, several proteins such as integrin, beta-catenin, nectin, otogelin, and shroom3 belong to adhesive components [12,13,14], which are potentially important factors for host cell attachment and infection progression during IBV infection.

A previous study identified heat shock protein 70 (HSP70) from the lungs and kidneys of IBV-infected chickens as a binding-associated protein to IBV S1 of the SCZJ3 strain, and infection of CEK cells by SCZJ3 was found to be inhibited by anti-HSP70 antibodies, indicating that HSP70 is a part of the IBV receptor complex [15]. HSP70 translocates to the membrane of host cells under certain conditions such as heat stress [16], tumor [17], or viral infection [18]. There is evidence that HSP70 serves as a receptor for several viruses [18,19,20]. In our current mass spectrometry data, we also identified HSP70 as a predicted binding-associated protein to S1 of YC181031. Importantly, immunoprecipitation and Western blot analyses showed that the IBV S1 protein of YC181031 is able to interact with HSP70 in TOCs and 293T cells (Figure 5).

We further investigated the effects of HSP70 on IBV replication by using knockdown and overexpression assays. However, we could not detect clear changes in HSP70 expression by transient transfection of HSP70 interference or overexpression plasmids using the TOC system (data not shown). Additional factors present in TOC may influence the knockdown and overexpression of host genes. In addition, due to the high inherent expression level of native HSP70 in the trachea organ, it may not be easy to interfere with its expression or to observe the effects of HSP70 overexpression upon virus infection. Further fine-tuning or modification of reaction conditions is required in order to use the TOC model to study the functions of host proteins on IBV infection.

Instead of using TOC, in Vero cells, we observed that HSP70 overexpression inhibited IBV Beaudette growth, indicating that HSP70 might be a potential host anti-viral factor for IBV infection (Figure 6). This result seems contradictory to a previous report where HSP70 was part of the receptor complex of IBV [15]. However, HSPs are multifunctional proteins, although the biological functions of HSPs include the maintenance of cellular homeostasis. In cases of viral infection, they can also be employed by viruses to correctly fold their proteins, assist the viruses in completing their life cycles at various stages, help the viruses adapt to and escape from antiviral responses of the host cells, or can be used by the host to combat viral infection, etc. [7]. We speculate that in the early stage of IBV infection, host HSP70 might assist virus attachment and entry into host cells while inhibiting virus replication after its invasion. The molecular mechanisms of HSP70 in IBV infection will continue to be investigated by our group in the near future.

## 5. Conclusions

In conclusion, we evaluated TOCs as an ex vivo culture model for infections with various IBV strains and carried out a study on IBV virus-host protein interaction. We found that all five selected different genotype IBV strains could replicate in the epithelial cells of the trachea of embryonated chicken eggs and gave rise to ciliostasis, loss of cilia, and cellular necrosis. A total of 127 predicted IBV S1-interacting host proteins were identified in infected TOCs, including interesting candidates potentially related to IBV invasion. Among them, HSP70 was further confirmed to interact with IBV S1 protein in both TOCs and transfected cells, an interaction that has potential use in antiviral therapy.

## Data Availability

Available in section “MDPI Research Data Policies” at https://www.mdpi.com/ethics.

## Supplementary Materials

The following supporting information can be downloaded at: https://www.mdpi.com/article/10.3390/v15051216/s1, Table S1: Candidate host proteins interacting with IBV S1; Table S2: The GO annotation analyses of IBV S1-interacting host proteins; Table S3: The KEGG enrichment analyses of IBV S1-interacting host proteins.

## References

[1] Jackwood M.W. (2012). Review of infectious bronchitis virus around the world. Avian Dis..

[2] Xu L., Han Z., Jiang L., Sun J., Zhao Y., Liu S. (2018). Genetic diversity of avian infectious bronchitis virus in China in recent years. Infect. Genet. Evol..

[3] Belouzard S., Millet J.K., Licitra B.N., Whittaker G.R. (2012). Mechanisms of coronavirus cell entry mediated by the viral spike protein. Viruses.

[4] Raj G.D., Jones R.C. (1996). An in vitro comparison of the virulence of seven strains of infectious bronchitis virus using tracheal and oviduct organ cultures. Avian Pathol..

[5] Maier H.J., Neuman B.W., Bickerton E., Keep S.M., Alrashedi H., Hall R., Britton P. (2016). Extensive coronavirus-induced membrane rearrangements are not a determinant of pathogenicity. Sci. Rep..

[6] Chhabra R., Kuchipudi S.V., Chantrey J., Ganapathy K. (2016). Pathogenicity and tissue tropism of infectious bronchitis virus is associated with elevated apoptosis and innate immune responses. Virology.

[7] Lubkowska A., Pluta W., Strońska A., Lalko A. (2021). Role of Heat Shock Proteins (HSP70 and HSP90) in Viral Infection. Int. J. Mol..

[8] Pujhari S., Brustolin M., Macias V.M., Nissly R.H., Nomura M., Kuchipudi S.V., Rasgon J.L. (2019). Heat shock protein 70 (Hsp70) mediates Zika virus entry, replication, and egress from host cells. Emerg. Microbes Infect..

[9] Xu T., Lin Z., Wang C., Li Y., Xia Y., Zhao M., Hua L., Chen Y., Guo M., Zhu B. (2019). Heat shock protein 70 as a supplementary receptor facilitates enterovirus 71 infections in vitro. Microb. Pathog..

[10] Manzoor R., Kuroda K., Yoshida R., Tsuda Y., Fujikura D., Miyamoto H., Kajihara M., Kida H., Takada A. (2014). Heat shock protein 70 modulates influenza A virus polymerase activity. J. Biol. Chem..

[11] Valastro V., Holmes E.C., Britton P., Fusaro A., Jackwood M.W., Cattoli G., Monne I. (2016). S1 gene-based phylogeny of infectious bronchitis virus: An attempt to harmonize virus classification. Infect. Genet. Evol..

[12] Wang S., Zhang Q., Tiwari S.K., Lichinchi G., Yau E.H., Hui H., Li W., Furnari F., Rana T.M. (2020). Integrin αvβ5 Internalizes Zika Virus during Neural Stem Cells Infection and Provides a Promising Target for Antiviral Therapy. Cell. Rep..

[13] Chatterjee S., Keshry S.S., Ghosh S., Ray A., Chattopadhyay S. (2022). Versatile β-Catenin Is Crucial for SARS-CoV-2 Infection. Microbiol. Spectr..

[14] Laksono B.M., de Vries R.D., McQuaid S., Duprex W.P., de Swart R.L. (2016). Measles Virus Host Invasion and Pathogenesis. Viruses.

[15] Zhang Z., Yang X., Xu P., Wu X., Zhou L., Wang H. (2017). Heat shock protein 70 in lung and kidney of specific-pathogen-free chickens is a receptor-associated protein that interacts with the binding domain of the spike protein of infectious bronchitis virus. Arch. Virol..

[16] Horváth I., Multhoff G., Sonnleitner A., Vígh L. (2008). Membrane-associated stress proteins: More than simply chaperones. Biochim. Biophys. Acta.

[17] Hantschel M., Pfister K., Jordan A., Scholz R., Andreesen R., Schmitz G., Schmetzer H., Hiddemann W., Multhoff G. (2000). Hsp70 plasma membrane expression on primary tumor biopsy material and bone marrow of leukemic patients. Cell Stress Chaperones.

[18] Reyes-Del Valle J., Chávez-Salinas S., Medina F., Del Angel R.M. (2005). Heat shock protein 90 and heat shock protein 70 are components of dengue virus receptor complex in human cells. J. Virol..

[19] Das S., Laxminarayana S.V., Chandra N., Ravi V., Desai A. (2009). Heat shock protein 70 on Neuro2a cells is a putative receptor for Japanese encephalitis virus. Virology.

[20] Broquet A.H., Lenoir C., Gardet A., Sapin C., Chwetzoff S., Jouniaux A.M., Lopez S., Trugnan G., Bachelet M., Thomas G. (2007). Hsp70 negatively controls rotavirus protein bioavailability in caco-2 cells infected by the rotavirus RF strain. J. Virol..

