# Supplementary material for: Identification of Host Proteins Interacting with IBV S1 Based on Tracheal Organ Culture
Source: Viruses. 2023 May 22;15(5):1216. doi: 10.3390/v15051216 (PMC10221944; doi:10.3390/v15051216)
Supplement: Supplementary file 1 [file viruses-15-01216-s001.zip › viruses-2349698-supplementary.pdf]

Supplementary Table S1 Candidate host proteins interacting with IBV S1

No.	Name of the proteins	Gene names
1	catenin beta-1	CTNNB1
2	microtubule-associated serine/threonine-protein kinase 4	MAST4
3	F-box/LRR-repeat protein 2	FBXL2
4	zinc finger protein 302	ZNF302
5	nucleolar protein 8	NOL8
6	anchorin CII	/
7	annexin A5	ANXA5
8	vacuolar H+-ATPase B	ATP6V1B2
9	Rho-associated coiled-coil forming kinase 1	Rock1
10	HSP 70	HSP70
11	MTGR1	MTGR1
12	CBFA2T2	CBFA2T2
13	Iron responsive element binding protein	IREB2
14	cytoplasmic aconitate hydratase	/
15	Hyperion protein	/
16	DEC-205 protein precursor	/
17	lymphocyte antigen 75 precursor	/
18	probable ATP-dependent RNA helicase DDX52	DDX52
19	oral-facial-digital syndrome 1	OFD1
20	potassium channel subfamily K member 5	KCNK5
21	HID1	HID1
22	integrin beta-4	ITGB4
23	cleavage stimulation factor subunit 3	CSTF3
24	flotillin-2	FLOT2
25	hematopoietic lineage cell-specific protein	LOC107055808
26	synaptotagmin-like protein 1	SYTL1
27	probable proline--tRNA ligase, mitochondrial precursor	PARS2
28	sodium voltage-gated channel alpha subunit 11	SCN11A
29	rab5 GDP/GTP exchange factor	RABGEF1
30	histone acetyltransferase type B catalytic subunit	HAT1
31	centromere protein F	CENPF
32	hemoglobin subunit alpha-A	HBA1
33	alpha-globin	HBA
34	Thyroid hormone receptor alpha	THRA
35	protein c-erbA	CERBA
36	Nuclear factor NF-kappa-B p100 subunit;	NFKB2
37	UAP56-interacting factor	/
38	rho GTPase-activating protein 26	ARHGAP26
39	ubiquitin-conjugating enzyme E2 T	UBE2T
40	chondroitin sulfate proteoglycan 4	CSPG4
41	cocaine- and amphetamine-regulated transcript protein	CART
42	WW domain-binding protein 11	WBP11

43	nectin-3	NECTIN3
44	cAMP-regulated phosphoprotein 21	ARPP21
45	unconventional myosin-Ie	MYO1E
46	nuclear factor interleukin-3-regulated protein	NFIL3
47	aminopeptidase O	AOPEP
48	bromodomain-containing protein 4	BRD4
49	metastasis-associated protein MTA2	MTA2
50	metallo-beta-lactamase domain-containing protein 1	MBLAC1
51	cystic fibrosis transmembrane conductance regulator	CFTR
52	synaptonemal complex protein 2-like	SYCP2L
53	PDZ and LIM domain protein 5	PDLIM5
54	FAM13A	FAM13A
55	TNF-related apoptosis inducing ligand-like protein	TRAIL-LIKE
56	acetyl-coenzyme A synthetase 2-like, mitochondrial	/
57	SLIT and NTRK-like protein 4	SLITRK4
58	probable E3 ubiquitin-protein ligase HERC1	LOC107056784
59	solute carrier family 2, facilitated glucose transporter member 8	SLC2A8
60	glucose transporter type 8	/
61	putative Polycomb group protein ASXL3	ASXL3
62	eyes absent homolog 1	EYA1
63	echinoderm microtubule-associated protein-like 6	EML6
64	bcl-2-associated transcription factor 1	BCLAF1
65	DNA helicase MCM9	MCM9
66	FAM184A	FAM184A
67	sn1-specific diacylglycerol lipase alpha	DAGLA
68	growth arrest-specific protein 2	GAS2
69	otogelin	OTOG
70	zinc finger and BTB domain-containing protein 42	ZBTB42
71	FRA10AC1	FRA10AC1
72	partitioning defective 3 homolog B	PARD3B
73	histone H2A deubiquitinase MYSM1	MYSM1
74	scm-like with four MBT domains protein 1	LOC107056889
75	intraflagellar transport protein 122 homolog	IFT122
76	lysine-rich nucleolar protein 1	KNOP1
77	myocardin	MYOCD
78	calcium/calmodulin-dependent protein kinase kinase 1	CAMKK1
79	putative Polycomb group protein ASXL1	ASXL1
80	osteoclast stimulatory transmembrane protein	OCSTAMP
81	immunoglobulin superfamily member 3	IGSF3
82	ralBP1-associated Eps domain-containing protein 1	REPS1
83	protein moonraker	/
84	kinesin-like protein KIF12	KIF12
85	KIF17	KIF17
86	sodium/hydrogen exchanger 8	/

87	phospholipid transfer protein	PLTP
88	solute carrier family 25 member 44	SLC25A44
89	plexin-A2	PLXNA2
90	rho GTPase-activating protein 23	ARHGAP23
91	gamma-tubulin complex component 6	/
92	zinc finger protein 646-like	LOC101749845
93	basic salivary proline-rich protein 2-like	LOC112530182
94	integrin beta-8	ITGB8
95	thioredoxin, mitochondrial	LOC107055648
96	actin-binding LIM protein 1	ABLIM1
97	pericentrin	PCNT
98	RNA helicase Mov10l1	MOV10L1
99	ATP-dependent RNA helicase DDX52	DDX52
100	fumarylacetoacetate hydrolase domain-containing protein 2-like	LOC107049688
101	meiosis-specific nuclear structural protein 1	MNS1
102	telomere repeats-binding bouquet formation protein 1	TERB1
103	histone deacetylase 11	HDAC11
104	serine-protein kinase ATM	ATM
105	serine/threonine-protein kinase WNK2	WNK2
106	neuronal pentraxin receptor	NPTXR
107	DENN domain-containing protein 2A	DENND2A
108	ras GTPase-activating-like protein IQGAP1	IQGAP1
109	NADH dehydrogenase [ubiquinone] 1 beta subcomplex subunit 10	NDUFB10
110	rabankyrin-5	ANKFY1
111	carbonic anhydrase 4	CA4
112	40S ribosomal protein S16	RPS16
113	vitamin K-dependent protein S	LOC107055759
114	zinc finger protein 501	ZNF501
115	growth hormone-regulated TBC protein	GRTP1
116	RWD domain-containing protein 2B	RWDD2B
117	transcription initiation factor TFIID subunit 4	LOC415780
118	radial spoke head protein 3 homolog	RSPH3
119	inhibitor of Bruton tyrosine kinase	IBTK
120	Shroom3	SHROOM3
121	mitoferrin-2	LOC105195750
122	SEC23-interacting protein	SEC23IP
123	UV excision repair protein RAD23 homolog B	RAD23B
124	KIAA1257 homolog	KIAA1257
125	SYVN1	SYVN1
126	LOC420107	LOC420107
127	LOC423301	LOC423301

Supplementary Table S2 The GO annotation analyses of IBV S1-interacting host proteins

Term	ID	Input	Total	P-Value	Corrected P-Value
adherens junction	GO:0005912	6	80	8.85E-07	5.52E-04
apical junction complex	GO:0043296	3	15	4.04E-05	1.26E-02
chromosome, telomeric region	GO:0000781	3	26	1.75E-04	2.59E-02
transcription coactivator activity	GO:0003713	5	140	2.22E-04	2.59E-02
apical plasma membrane	GO:0016324	5	144	2.52E-04	2.59E-02
glomerular visceral epithelial cell development	GO:0072015	2	5	2.90E-04	2.59E-02
regulation of myoblast differetiation	GO:0045661	2	5	2.90E-04	2.59E-02
peroxisome proliferator activated receptor binding	GO:0042975	2	6	3.86E-04	2.80E-02
thymus development	GO:0048538	3	38	4.95E-04	2.80E-02
chloride channel inhibitor activtiy	GO:0019869	2	7	4.96E-04	2.80E-02
flotillin complex	GO:0016600	2	7	4.96E-04	2.80E-02
smooth muscle cell differentiation	GO:0051145	2	8	6.18E-04	2.80E-02
oocyte development	GO:0048599	2	8	6.18E-04	2.80E-02
vasculogenesis	GO:0001570	3	44	7.41E-04	2.80E-02
ATP binding	GO:0005524	12	1073	7.48E-04	2.80E-02
lens morphogenesis in camera-type eye	GO:0002089	2	9	7.53E-04	2.80E-02
positive regulation of transcription by RNA polymerase II	GO:0045944	10	779	7.63E-04	2.80E-02
chromatin binding	GO:0003682	6	300	1.06E-03	3.66E-02

Supplementary Table S3 The KEGG enrichment analyses of IBV S1-interacting host proteins

Term	Input	Total	P-Value	Corrected P-Value
ECM-receptor interaction	2	83	4.09E-02	4.38E-01
Focal adhesion	3	189	3.60E-02	4.38E-01
Regulation of actin cytoskeleton	2	190	1.63E-01	4.81E-01
Nucleotide excision repair	1	38	1.37E-01	4.73E-01
Protein processing in endoplasmic reticulum	2	153	1.16E-01	4.73E-01
Ubiquitin mediated proteolysis	1	127	3.84E-01	4.93E-01
Adhesion junciton	2	70	3.02E-02	4.38E-01
Melanogenesis	1	91	2.94E-01	4.93E-01
Wnt signaling pathway	1	141	4.15E-01	4.93E-01
ABC transporters	1	40	1.43E-01	4.73E-01
Tight junction	1	156	4.48E-01	4.93E-01
Homologous recombination	1	38	1.37E-01	4.73E-01
p53 signaling pathway	1	66	2.23E-01	4.93E-01
Cell cycle	1	114	3.52E-01	4.93E-01
FoxO signaling pathway	1	123	3.74E-01	4.93E-01
Apoptosis	1	127	3.84E-01	4.93E-01
Oxidative phosphorylation	2	113	6.99E-02	4.38E-01
Phagosome	1	132	3.95E-01	4.93E-01
mTOR signaling pathway	1	140	4.13E-01	4.93E-01
Metabolic pathway	3	1290	8.71E-01	8.71E-01
C-type lectin receptor signaling pathway	1	87	2.83E-01	4.93E-01
MAPK signaling pathway	1	261	6.30E-01	6.70E-01
Nitrogen metabolism	1	17	6.56E-02	4.38E-01
Fanconi anemia pathway	1	50	1.63E-01	4.81E-01
Cell adhesion molecules（CAMs）	2	122	7.95E-02	4.38E-01
PPAR signaling pathway	1	64	2.18E-01	4.93E-01
mRNA surveillance pathway	1	70	2.35E-01	4.93E-01
